# Establishment and Validation of a 5 m6A RNA Methylation Regulatory Gene Prognostic Model in Low-Grade Glioma

**DOI:** 10.3389/fgene.2022.655169

**Published:** 2022-02-25

**Authors:** Zhiqun Bai, Xuemei Wang, Zhen Zhang

**Affiliations:** Department of Ultrosonic Diagnosis, The First Affiliated Hospital of China Medical University, Shenyang, China

**Keywords:** LASSO, M6A RNA methylation, LGG, prognostic model, ICI

## Abstract

**Background:** The prognosis of low-grade glioma (LGG) is different from that of other intracranial tumors. Although many markers of LGG have been established, few are used in clinical practice. M6A methylation significantly affects the biological behavior of LGG tumors. Therefore, establishment of an LGG prognostic model based on m6A methylation regulatory genes is of great interest.

**Methods:** Data from 495 patients from The Cancer Genome Atlas (TCGA) and 172 patients from the Chinese Glioma Genome Atlas (CGGA) were analyzed. Univariate Cox analysis was used to identify methylation regulatory genes with prognostic significance. LASSO Cox regression was used to identify prognostic genes. Receiver operating characteristic (ROC) and Kaplan–Meier curves were used to verify the accuracy of the model. Gene Set Enrichment Analysis (GSEA) and the Kyoto Encyclopedia of Genes and Genomes (KEGG) were used to identify cellular pathways that were significantly associated with the prognosis of LGG.

**Results:** A glioma prognostic model based on five methylation regulatory genes was established. Compared with low-risk patients, patients identified as high risk had a poorer prognosis. There was a high degree of consistency between the internal training and internal validation CGGA cohorts and the external validation TCGA cohort. Furthermore, KEGG and GSEA analyses showed that the focal adhesion and cell cycle pathways were significantly upregulated in high-risk patients. This signature could be used to distinguish among patients with different immune checkpoint gene expression levels, which may inform immune checkpoint inhibitor (ICI) immunotherapy.

**Conclusion:** We comprehensively evaluated m6A methylation regulatory genes in LGG and constructed a prognostic model based on m6A methylation, which may improve prognostic prediction for patients with LGG.

## Introduction

Low-grade gliomas (II and III of the World Health Organization) are the most common primary malignant tumors in the brain and are mainly localized to the cerebral hemispheres (Ostrom et al., 2013). Neurosurgery, radiotherapy, and chemotherapy are common treatment strategies, but recurrence and drug resistance rates are high (Brat et al., 2015; Hayes et al., 2018). Some patients will quickly develop high-grade glioblastoma, resulting in a very poor prognosis (Zhang et al., 2016). Therefore, it is urgent to identify a sensitive and accurate biomarker to predict the prognosis of patients with LGG.

N6-methyladenosine (m6A) is the most studied RNA modification (Desrosiers et al., 1974), which plays an important role in the posttranscriptional regulation of RNA in eukaryotes (Zhao et al., 2017). The m6A modifications can occur on both mRNA and ncRNA (Alarcón et al., 2015). When m6a modifications occur in mRNA, they play multiple roles in mRNA processing and metabolism, including splicing, stability, nuclear export, and translation (Lee et al., 2020). In addition, m6A modification is also found in most ncRNAs, including miRNA, lncRNA, and circRNA, which participated in multiple roles in chromatin remodeling, transcription, posttranscriptional modifications, and signal transduction (Yang et al., 2020). M6A is dynamically and reversibly regulated by an m6A regulator, including methyltransferases (writers), demethylases (erasers), and binding proteins (readers) to add, remove, or recognize m6A-modified sites, respectively, thereby altering important biological processes accordingly (Guo et al., 2021). The m6A methyltransferases (writers) mediate the process of methylation modification of RNA, which mainly includes methyltransferase-like 3 (METTL3), methyltransferase-like 14 (METTL14), and Wilms’ tumor 1-associating protein (WTAP) (Meyer and Jaffrey, 2017). The m6A demethylases (erasers) including obesity-associated protein (FTO) and alkB homolog 5 (ALKBH5), which mediate the process of decreasing m6A modifications of RNA, are the key to the reversibility of the m6A modification process (Meyer and Jaffrey, 2017). The m6A binding proteins (readers) are able to specifically recognize m6A-modified RNAs and participated in the regulation of RNA splicing, turnover, export, and translation and m6A readers mainly including YTH domain family YTHDF1-3, YTHDC1-2, insulin-like growth factor 2, mRNA-binding proteins IGF2BP1-3, heterogeneous nuclear ribonucleoprotein A2B1 (HNRNPA2B1), heterogeneous nuclear ribonucleoprotein C (HNRNPC), and embryonic lethal abnormal vision *Drosophila* like 1 (ELAVL1) (Meyer and Jaffrey, 2017). M6A, as a modification in RNA, plays an important role in bioprocesses such as self-renewal and differentiation of embryonic stem cells and hematopoietic stem cell, tissue development, circadian rhythm, heat shock or DNA damage response, and sex determination, although it does not change base pairing and coding (Huang et al., 2020). To investigate the specific mechanisms of m6a in cells or tissues, the expression of m6A regulatory genes, the global m6A abundance in RNA, and m6A modification site and gene need to be detected (Wang and Jia, 2020). LC-MS/MS, 2D-TLC, and dot blot can be used to detect the global m6A abundance in RNA. MeRIP-qRT-PCR, MeRIP-seq, SCARLET, SELECT, the m6A-sensitive deoxyribozyme method, the m6A-sensitive base-pairing method, and m6A-sensitive HRM analysis can be used to detect the m6A modification site and gene (Wang and Jia, 2020). Meanwhile, several databases, including RMBase (Xuan et al., 2018), MeT-DB (Liu H et al., 2018), CVm6A (Han et al., 2019), RNAmod (Liu and Gregory, 2019), SRAMP (Zhou et al., 2016), REPIC (Liu et al., 2020), and M6ADD (Zhou et al., 2021) were constructed to organize and integrate existing resources in order to better explore the mechanism of m6A.

Numerous studies have shown that the global abundance of m6A and the expression levels of m6A regulatory genes, which are frequently dysregulated in various types of cancer, are critical for cancer development, progression, and metastasis, as well as drug resistance and cancer recurrence (Huang et al., 2020). Decreases or increases in global m6A abundance have recently been reported in several cancer types that may be associated with cancer progression and clinical outcomes (Huang et al., 2020). The global m6A abundance was aberrantly upregulated in gastric cancer (Wang J et al., 2020), while aberrantly downregulated in bladder cancer (Gu et al., 2019). In addition, abnormalities in m6A regulatory genes can lead to a range of diseases, including cancer, neurological disorders, embryonic developmental delays, and obesity (Jiang et al., 2021). Studies have shown that M6A modifications plays important roles in various tumors and are involved in tumor proliferation, carcinogenesis, and migration (Meyer et al., 2012; Ke et al., 2015). For example, in glioblastoma multiforme (GBM), METTL3 suppresses the proliferation and self-renewal of glioblastoma stem cells by enhancing m6A modification of ADAM19 and decreasing its expression, which suppressed the progression of GMB (Visvanathan et al., 2018). METTL3 and FTO3 play an oncogenic role in acute myeloid leukemia (Vu et al., 2017). Some studies have indicated that genes that express methylation enzymes, including YTHDC2, RBM15B, METTL16, YTHDF3, IGF2BP3, RBM15, METTL14, ZC3H13, YTHDF1, YTHDF2, ALKBH5, HNRNPA2B1, ALKBH3, IGF2BP1, HNRNPC, YTHDC2, METTL3, WTAP, YTHDC1, IGF2BP2, and FTO (Ma et al., 2021), may be important in LGG (Tu et al., 2020; Fang et al., 2021; Kowalski-Chauvel et al., 2020; Yarmishyn et al., 2020). However, the expression status of a single m6A regulatory gene is not sufficient to describe a patient’s status and outcome.

**FIGURE 1:**
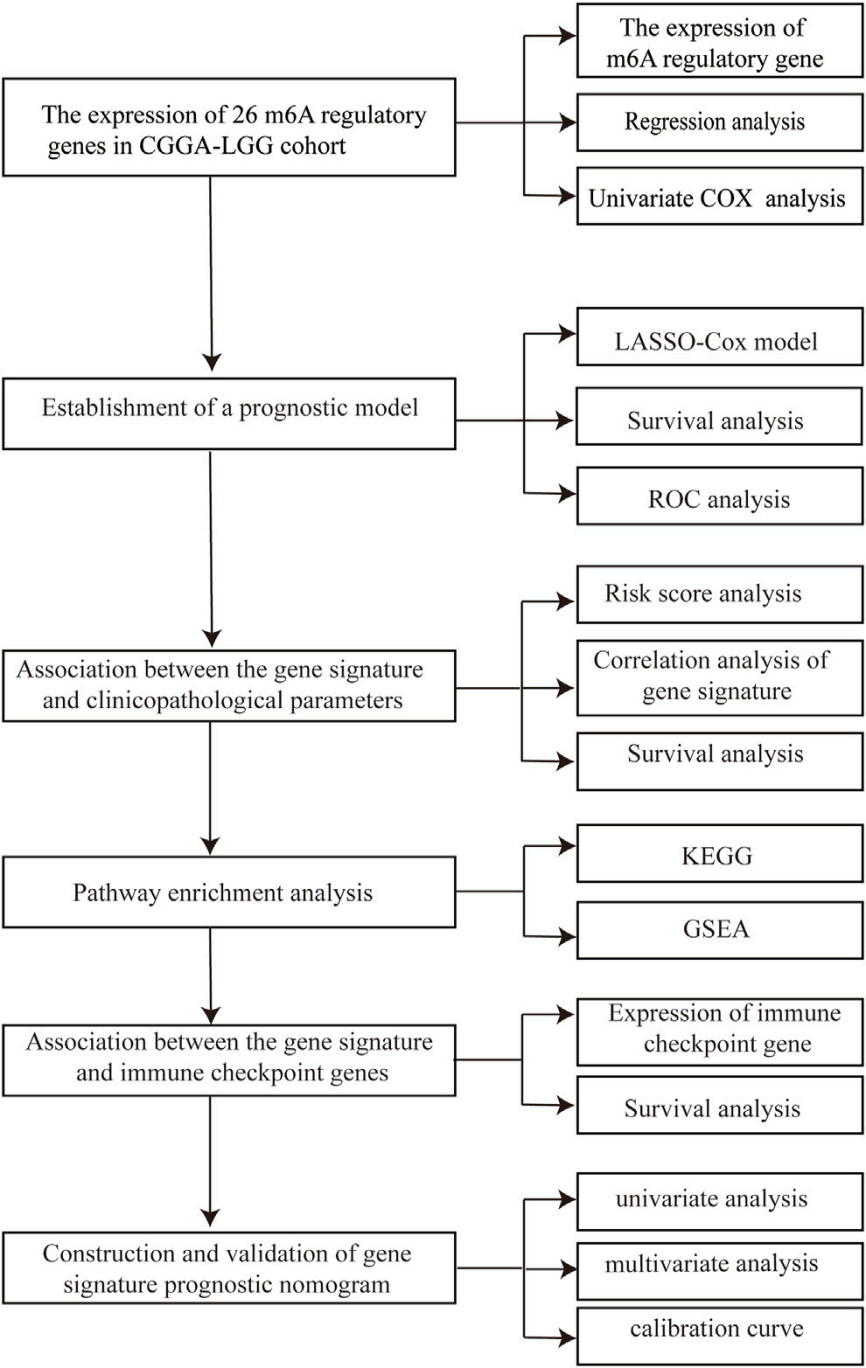
Flow chart of the analysis methods utilized in the current study.

In this study, we systematically characterized the expression levels of a group of m6A methylation regulator genes in patients with LGG and constructed a prognostic model for LGG. As a result, we established a framework to quantify prognosis using an integrated analysis of the expression status of 5 m6A methylation regulatory genes, which resulted in a robust approach to prediction of overall survival (OS). The main flow of the article can be found in Figure 1. This approach, using a novel gene expression signature, is promising as a predictor OS of LGG.

## Materials and Methods

### Data Preprocessing

Gene expression profiles and survival data for patients with LGG were downloaded from the CGGA (http://www.cgga.org.cn/) database. Overall, 495 samples from TCGA and 172 samples from the CGGA were analyzed (Table 1). Perl and R in R software were utilized to evaluate the samples.

**TABLE 1:** Two low-grade glioma datasets used in this study.

Datasets	Platform	Component of samples	Use
TCGA LGG mRNA-seq	IlluminaHiseq_RNAseq	534 lower-grade glioma	Internal training and validation set for prognositc gene signature
CGGA mRNA-seq_325	Illumina Hiseq 2000	182 lower-grade glioma	External validation set for prognositc gene signature

### Identifying the m6A RNA Methylation Regulatory Gene in LGG

The expression level of a total of 26 m6A RNA methylation regulatory genes in LGG samples and normal samples in TCGA and CGTA datasets were detected by the R package “limma” and visualized by the R package “pheatmap.” The correlations between 26 m6A RNA methylation regulatory genes were detected by the R package “corrplot.”

### Establishment of the LASSO Cox Signature

A total of 172 patients from the CGGA database were randomly assigned in a 1:1 ratio to a training set and a validation set. The hazard ratio of OS of 26 m6A regulatory genes in the internal training set was calculated using the univariate Cox proportional hazard regression model, and genes with *p* < 0.05 were considered statistically significant and included in subsequent analyses. Then, m6A-related genes that were identified as significant in the univariate analysis in the internal training set were included in the penalized Cox regression model with the least absolute shrinkage and selection operator (LASSO) Cox regression model for 10-fold cross validations to select the most significant genes. Finally, an m6A-related prognostic gene signature was constructed based on a linear combination of the regression coefficient derived from the LASSO Cox regression model coefficients multiplied by the mRNA expression level (Tibshirani,^1997^).

### Risk Score Evaluation and Survival Analysis

The risk score was calculated as follows: ∑Coefi × Expi , where Coefi is the coefficient of prognostic biomarker and Expi is the expression of the corresponding prognostic biomarker. According to the median value of the risk score, LGG patients in the CGGA cohort were divided into low-risk and high-risk groups. The overall Kaplan–Meier survival curves of low-risk and high-risk patients were analyzed by the R package “survival” and “survminer.”

### ROC Curve

The receiver operating characteristic curve (ROC) analysis was used to assess the accuracy of the diagnostic gene signature. The R software package “pROC” was used to generate an ROC curve (Robin et al., 2011). ROC area under curve (AUC) was calculated to evaluate the performance of each signature, and AUC>0.7 was considered as a diagnostic gene signature.

### Pathway Enrichment Analysis

The Kyoto Encyclopedia of Genes and Genomes (KEGG) analysis led to identification of enrichment pathways, which help to determine the biological pathways to which the identified genes belonged (Yu et al., 2012). The KEGG analysis is performed by the R package “clusterProfiler.”

### Gene Set Enrichment Analysis

Gene Set Enrichment Analysis v2.2.2 (http://www.broadinstitute.org/gsea) was used to investigate the biological differences among patients with different expression patterns of the 5-gene signature. In addition, C2:CP KEGG gene sets from the Molecular Signatures database (MSigDB) were used as the reference gene sets.

### Construction and Validation a Predictive Nomogram

The gene signatures were used to construct a nomogram by the “rms” R package, and the accuracy of the nomogram was evaluated by the calibration curve (1,000 bootstrap resamples).

### Statistical Analysis

Continuous variables were analyzed using Student’s t-tests or non-parametric tests. Categorical variables were analyzed using chi-squared tests or Fisher’s exact tests. The R package copy was used for univariate and multivariate analyses. All data were analyzed using SPSS or R software (http://www.r-project.org/). *p* < 0.05 was considered statistically significant.

## Results

### m6A Regulatory Gene Profile in the CGGA Cohort

**FIGURE 2:**
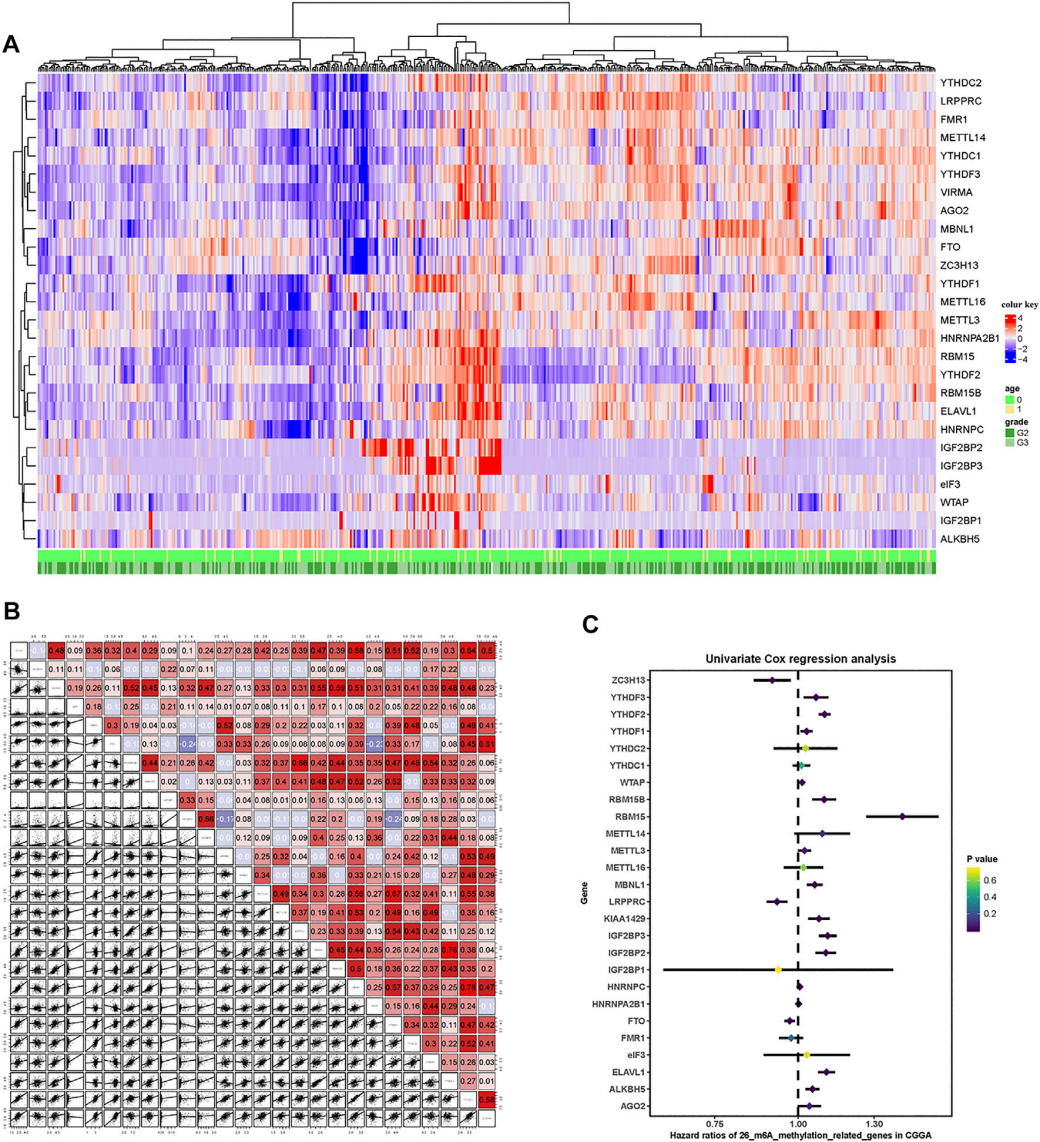
m6A-related gene profile in the CGGA cohort. **(A)** Unsupervised clustering of patients with LGG from the CGGA cohort using 26 m6A methylation regulatory genes. The red cube represents highly expressed genes, and the blue cube represents genes with lower expression levels. **(B)** Correlation of the 26 m6A methylation regulatory genes. **(C)** Forest plots showing associations between different m6A methylation regulatory genes and OS in the internal training CGGA cohort.

A total of 26 m6A RNA methylation regulator genes were included in this study to evaluate the methylation status of tumor tissues. We constructed a profile containing 26 m6A regulatory genes in patients with LGG. The m6A regulatory genes and corresponding clinical parameters are shown in Figure 2A. As shown by the heatmap, the expression levels of the m6A regulatory genes differed among the patients. To better understand the relationships among m6A regulatory genes in LGG, we performed a regression analysis of the m6A regulatory genes (Figure 2B). The results showed that YTHDF2 was highly correlated with RBM15. In addition, there was a strong correlation between VIRMA and YTHDF3. These results indicated that these pairs of genes may be involved in the same biological functions. To determine which m6A regulatory genes had prognostic value, we conducted a univariate COX analysis. This analysis identified 16 of the 26 methylated genes as prognostic indicators (Figure 2C).

### Establishment of a Prognostic Model

**FIGURE 3:**
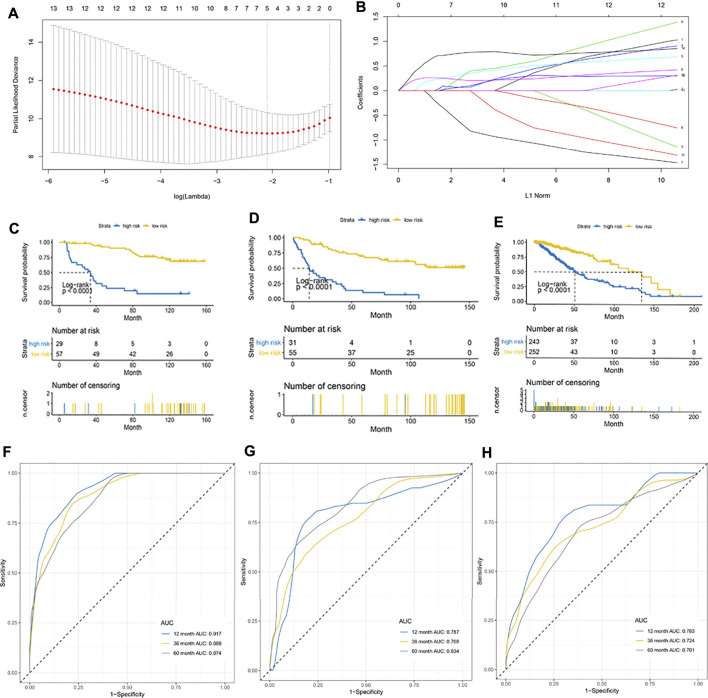
Establishment of a 5-gene prognostic model. **(A)** LASSO coefficient profiles of the fractions of immune cells. The minimum lambda value was reached when the number of genes was 5. **(B)** Parameter selection for tuning by 10-fold cross validation in the LASSO model. **(C–E)** Kaplan–Meier curve for patients with high and low risk in the internal training CGGA cohort, internal validation CGGA cohort, and external validation TCGA cohort, respectively. **(F–H)** Risk score measured using time-dependent receiver operating characteristic (ROC) curves in the internal training cohort, internal validation CGGA cohort, and external validation TCGA cohort at 1, 3, and 5 years, respectively.

A total of 172 patients in the CGGA cohort were randomly divided in a 1:1 ratio into an internal training cohort and a validation cohort. The 16 m6A regulatory genes with prognostic value were included in the LASSO Cox model in the internal training CGGA cohort to screen for characteristic variables (Figures 3A,B). As shown in Figure 2A, the minimum lambda value was reached with inclusion of 5 genes. Therefore, we selected 5 genes to construct a prognostic model. Finally, we constructed the following formula for prognosis of OS in patients with LGG: (formula= (0.033 ×HNRNPC) + (0.237 × IGF2BP2) + (0.260 × IGF2BP3) − (0.271 × LRPPRC) + (0.713 × YTHDF2)). According to the cutoff value (2.03) obtained using the survminer package, the patients in the training cohort were divided into high- and low-PRI groups. Patients with high risk had more events and a worse prognosis (Figure 3C). Application of the cutoff value to the internal validation CCGA cohorts and external validation TCGA cohorts produced the same result as that observed in the training group (Figures 3D,E). To evaluate the accuracy of this 5-gene signature for determination of prognosis, we generated an ROC curve. In the internal training cohort, the accuracy of the 5-gene signature was investigated at 2, 3, and 5 years, which resulted in AUC values of 0.917, 0.889, and 0.874, respectively (Figure 3F). In the internal validation cohort, the AUC values were 0.787, 0.768, and 0.834, respectively (Figure 3G). In the external validation TCGA cohort, the AUC values at 2, 3, and 5 years were 0.783, 0.721, and 0.701, respectively (Figure 3H). In summary, the 5-gene profile was an excellent prognostic indicator for patients with LGG.

### Association Between the 5-Gene Signature and Clinicopathological Parameters

**FIGURE 4:**
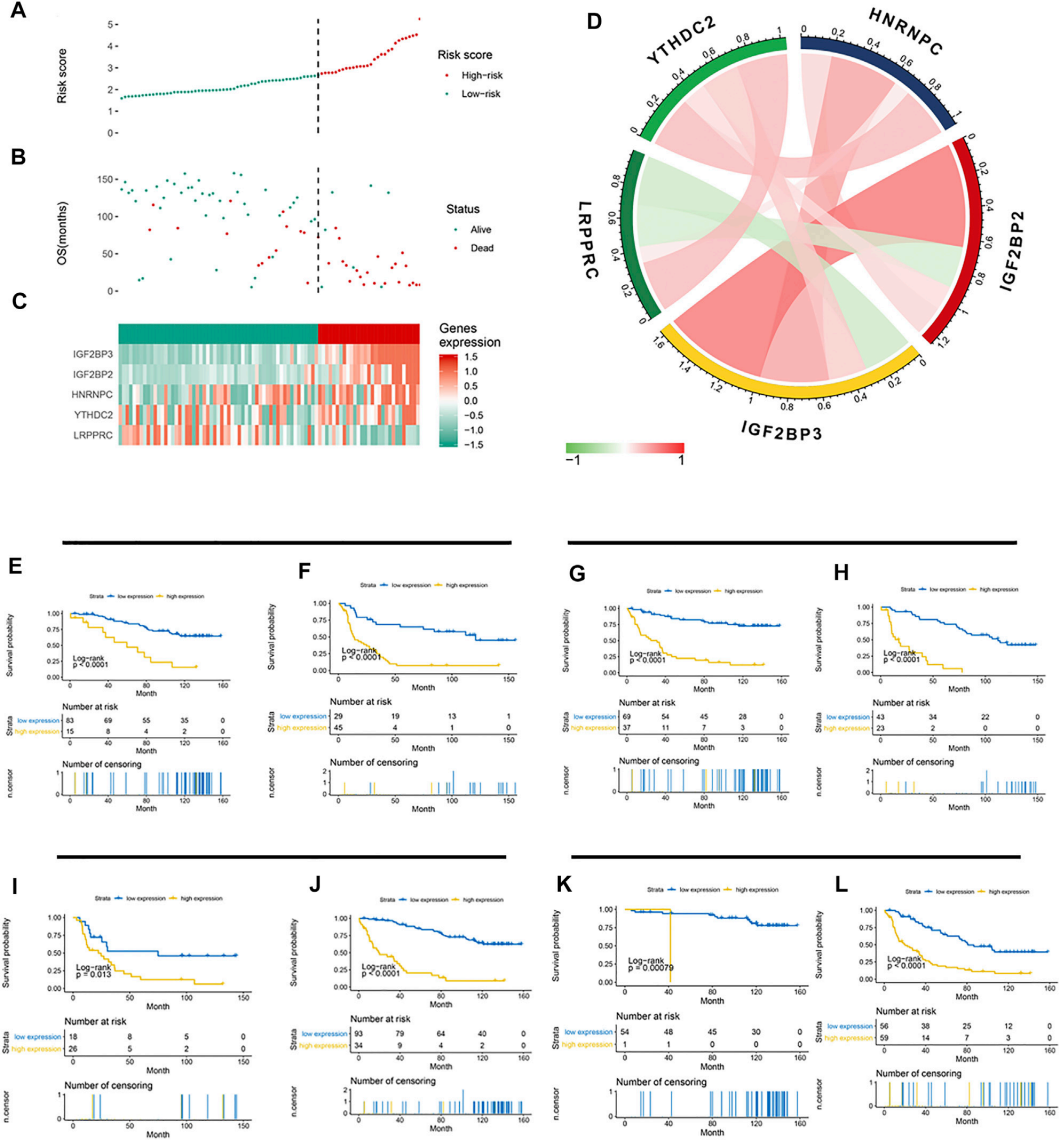
Association between the 5-gene signature and clinicopathological parameters. **(A–C)** 5-gene signature-based risk score in the CGGA cohort. **(A)** Risk score plot based on the 5-gene signature. **(B)** Live/dead state corresponding to the risk score in the upper panel. **(C)** Z-score-transformed expression value of each gene in the 5-gene signature. **(D)** Correlation analysis of the 5 methylation regulatory genes in the signature. **(E–L)** Kaplan–Meier curve showed significant statistical differences in overall survival between the high- and the low-risk groups regardless of gender **(E,F)**, WHO grade **(G,H)**, IDH mutation status **(I,J)**, and co-mutation state of chromosomes 1p and 19q **(K,L)**.

Based on the LASSO Cox model, the prognostic risk score for each patient in the CGGA cohort was calculated according to the factor value and expression level. According to the median risk score, patients were divided into a high-risk group and a low-risk group (Figures 4A–C). The high-risk group had higher levels of IGF2BP2, IGF2BP3, HNRNPC, and YTHDC2 and lower levels of LRPPRC. More patients survived in the low-risk group than in the high-risk group. Further correlation analysis found a negative correlation between LRPPRC and IGF2BP2 and between LRPPRC and IGF2BP2 (Figure 4D). We further evaluated the stability of 5-gene signature in different groups. The results showed that the 5-gene signature was an excellent prognostic indicator regardless of gender (Figures 4E,F), grade (Figures 4G,H), IDH mutation status (Figures 4I,J), and the co-mutation state of chromosome 1p and 19q (Figures 4K,L).

### Pathway Enrichment Analysis

**FIGURE 5:**
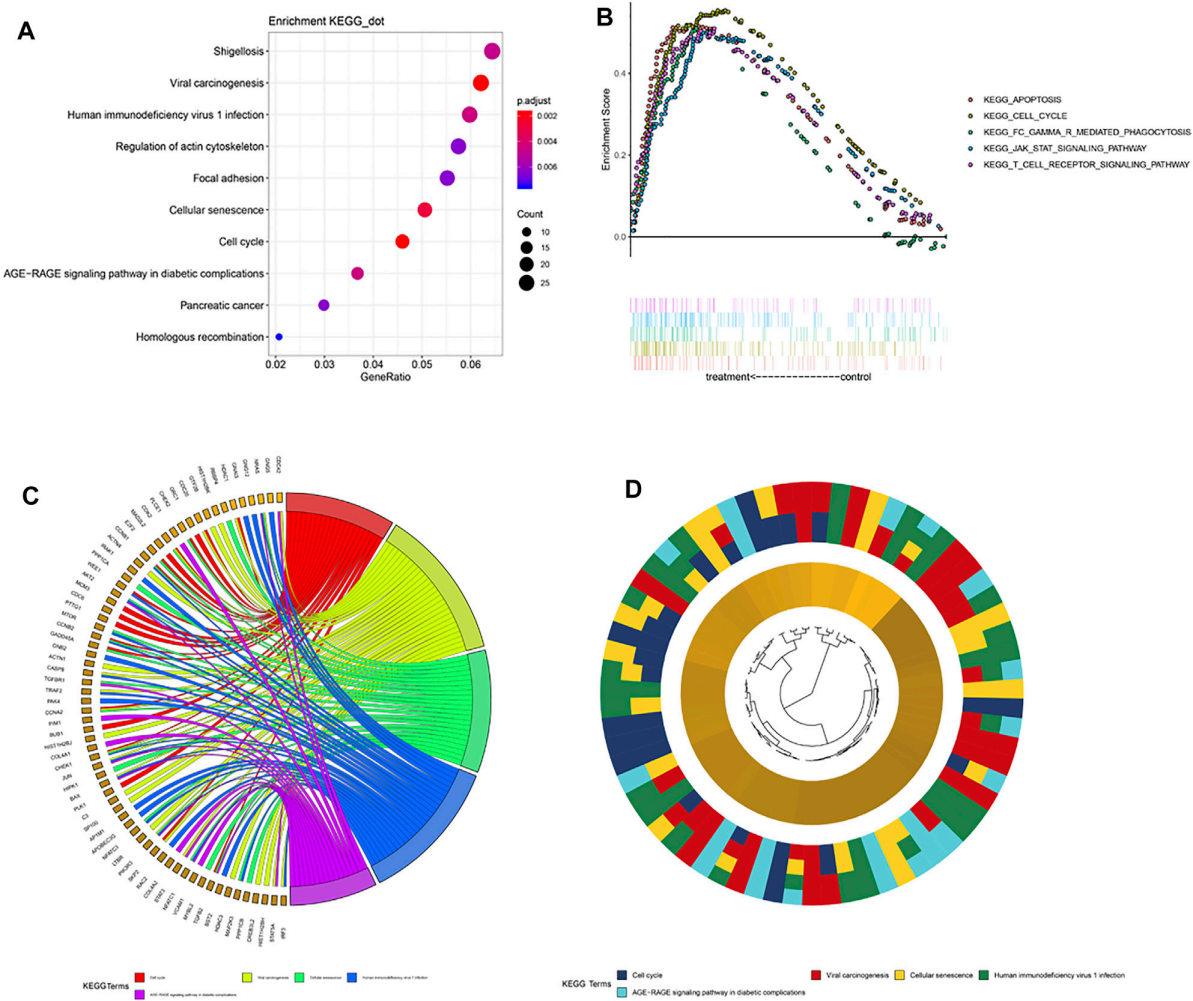
Pathway enrichment analysis. **(A)** Spearman correlation for PRI top 1,000 genes was used for KEGG analysis. These genes were enriched in KEGG pathways “cell cycle” and “focal adhesion.” **(B)** GSEA terms significantly enriched in the CGGA cohort. “KEGG_CELL_CYCLE,” “KEGG_APOPTOSIS,” “KEGG_JAK_STAT_SIGNALING_PATHWAY,” and “KEGG_T_CELL_RECEPTOR_SINGALING_PATHWAY” were significantly enriched. **(C)** Hierarchical clustering of gene expression profiles for each KEGG pathway. **(D)** Chord plots show the relationship between genes and the KEGG pathway.

To elucidate the differences in biological characteristics between the high- and low-risk groups using the 5-gene signature, we performed Spearman’s correlation analysis and selected the first 1,000 genes. Then, the clusterProfiler package in R software was used to perform KEGG enrichment analysis. These genes are significantly enriched in the focal adhesion and cell cycle pathways (Figure 5A). Specifically enriched genes for each KEGG term are summarized in Figures 5C,D. Next, we divided the patients into high- and low-risk groups according to their risk scores for the GSEA analysis, which showed enrichment of the 5-gene signature in cell cycle pathways (Figure 5B), which indicated that the cell cycle pathway may be a critical factor in poor prognosis in patients with LGG. In addition, expression levels of genes in the T-cell receptor signaling pathway were abnormally high in patients with LGG, which indicated that immune status may have differed with level of risk. Therefore, we further explored the expression levels of immune checkpoint (IC) genes in the different patient risk groups, which may provide additional information regarding personalization of treatment.

### Association Between the 5-Gene Signature and Immune Checkpoint Genes

**FIGURE 6:**
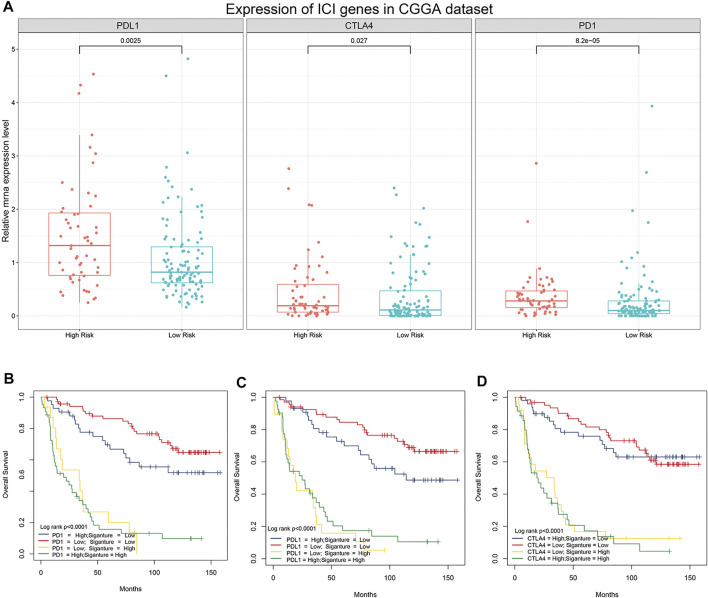
Association between the 5-gene signature and immune checkpoint genes. **(A)** Comparison of the expression pattern of immune checkpoint genes (PD-1, PD-L1, and CTLA-4) between patients with different risk scores in the CGGA analysis. **(B)** Kaplan–Meier survival curves of overall survival among four patient groups stratified by the risk score and PD-1 **(B)**, PD-L1 **(C),** and CTLA-4 **(D)**.

Previous studies have shown that the expression levels of immune checkpoint (IC) genes are associated with immunotherapy efficacy. We compared the expression patterns of IC genes (PD-1, PD-L1, and CTLA-4) in different risk groups of patients with LGG. As shown in Figure 6A, patients in the high-risk group had higher expression levels of IC genes. We further evaluated whether our research model for IC genes was generalizable to patients with similar expression levels of IC genes. As shown in Figure 6B, the overall survival of patients with low risk scores and high PD-1 expression was significantly better than that of patients with high risk scores and high PD-1 expression. Furthermore, the survival of patients with low risk scores and low PD-1 expression was longer than that of patients with high risk scores and low PD-1 expression. Similar results were observed for PD-L1 and CTLA-4 (Figures 6C,D). Stratification of patients with low risk scores according to IC gene expression showed that IC gene expression was significantly associated with survival of patients with low risk scores. However, there was no differences in survival of patients with high risk scores when stratified based on IC gene expression. In addition, patients with low risk scores and low IC gene expression tended to have much higher survival rates than patients in the other three groups. These results suggested that our 5-gene model may be a predictor of ICI immunotherapy efficacy.

### Construction and Validation of a 5-Gene Signature Prognostic Nomogram

**FIGURE 7:**
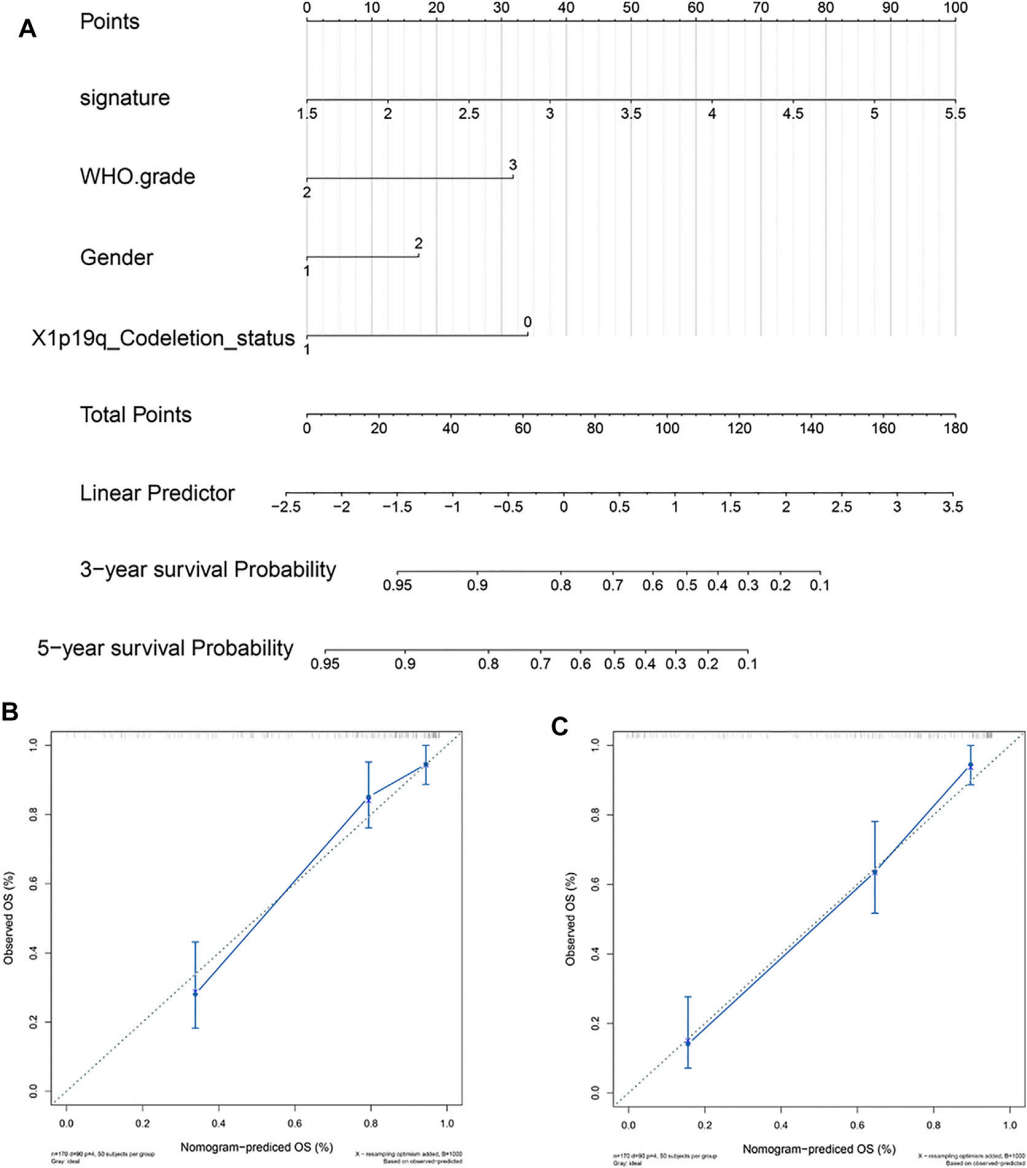
Construction and validation of a 5-gene signature prognostic nomogram. **(A)** Prognostic nomogram based on the 5-gene signature, WHO grade, gender, and X1p19q codeletion status. **(B,C)** Calibration curve at 3 and 5 years.

To provide patients with more accurate prognostic predictions, we incorporated the 5-gene signature and clinical parameters that had prognostic value in the univariate analysis and performed a multivariate analysis in the CGGA cohort. The results showed that the 5-gene signature, WHO grade, gender, and X1p19q codeletion status were stable predictors (Supplement Tables S1,2). These results were used to construct a nomogram to predict the prognosis of patients (Figure 7A). The calibration curve shows that the nomogram had stable predictive values at 3 and 5 years (Figures 7B,C).

## Discussion

Low-grade glioma (LGG) is a common invasive brain tumor in adults, and it includes World Health Organization (WHO) grade II and III diffuse gliomas (Ostrom et al., 2013). Although some progress has been made in the treatment of LGG with advances in neurosurgery, chemotherapy, and radiotherapy, a considerable number of patients experience recurrent and malignant glioblastoma multiforme (Chaichana et al., 2010; Okita et al., 2015; Deng et al., 2019; Fukuya et al., 2019; Chen et al., 2020; Mathur et al., 2020), resulting in decreased quality of life and shortened lifespan. The heterogeneity of prognosis for patients with LGG highlights the need to develop effective biomarkers for early stratification and preventive treatment of high-risk patients with poor prognoses.

M6A methylation, as the most widespread internal modification of mRNA, has been shown to play an important role in many cell types (Desrosiers et al., 1974; Dominissini et al., 2012; Fu et al., 2014; Wang et al., 2014; Ma et al., 2019; Sun et al., 2019). Many studies have shown that m6A regulatory genes can be used as markers to predict the prognosis of many kinds of cancers. (Liu J et al., 2018; Chen et al., 2019; Zhao and Cui 2019; Qu et al., 2020; Wang Q et al., 2020). Many previous studies have evaluated individual methylation regulatory genes, which may not be an accurate reflection of overall regulation of methylation. In this study, we evaluated multiple methylation regulatory genes in patients with LGG and selected a group of methylation regulatory genes that had prognostic value for patients with LGG through univariate Cox analysis and LASSO Cox modeling. Finally, a 5-gene signature was constructed (HNRNPC, IGF2BP2, IGF2BP3, LRPPRC, and YTHDF2) with good prognostic value and consistency between the internal validation and the external validation TCGA cohorts. To determine the pathways most closely associated with poor prognosis of patients with LGG, we performed a correlation analysis and selected the 1,000 genes most related to risk score. Furthermore, the KEGG enrichment analysis showed that differences in survival may have been associated with the cell cycle and focal adhesion pathways, which provides novel potential targets for treatment of LGG.

Advances in immune checkpoint inhibitor therapies have resulted in effective treatment of many cancers (Havel et al., 2019; Doroshow et al., 2019; Zhao et al., 2019; Gedeon et al., 2020). These advances have highlighted the importance of screening patients to determine whether they are good candidates for ICI treatment. The expression levels of independent immune checkpoint genes such as PD-L1 cannot be used as an independent predictor of ICI response (Gridelli et al., 2017; Lupo et al., 2018). By comparing the survival distribution of patients following stratification based on our 5-gene signature and immune checkpoint gene expression levels, we were able to show that our 5-gene profile correlated well with IC gene expression and risk, which indicated that our 5-gene signature can be used as a biomarker for knowing whether a patient is a good candidate for immunotherapy.

In summary, our study showed that m6A methylation regulatory genes could be used to classify patients with LGG into high- or low-risk subgroups exhibiting significantly different OS. Furthermore, this risk score may also be a marker for predicting the efficacy of ICI immunotherapy.

## Data Availability

The original contributions presented in the study are included in the article/Supplementary Material, further inquiries can be directed to the corresponding author.
